# Dynamic Determinants of the TLR4/MD2/Glycolipid Binding Unveiled Through Graph Neural Networks

**DOI:** 10.1002/cbic.70581

**Published:** 2026-11-14

**Authors:** Olmo Martín‐Cámara, Álvaro Arrayás‐Ruiz, Bruno Cuevas‐Zuviría, Sonsoles Martín‐Santamaría

**Affiliations:** ^1^ Centro de Investigaciones Biológicas Margarita Salas, CIB‐CSIC Madrid Spain; ^2^ Departamento de Biotecnología y Biología Vegetal Universidad Politécnica de Madrid Madrid Spain

**Keywords:** glycolipids, graph neural networks, ligand–protein interactions, molecular dynamics simulations, Toll‐like receptor 4

## Abstract

Molecular dynamics (MD) simulations provide insights about the molecular world, but only if analyzed properly. Determining the relevant dynamics in a protein–ligand system often becomes the main bottleneck of the analysis. Here, we analyze the performance of graph neural networks (GNNs) trained on the MD simulations of several complexes of the innate immunity receptor TLR4/MD2 with different glycolipids to disentangle the atomic/molecular characteristics that determine the agonist/antagonist activity. By systematically evaluating distinct atomic subselections, we found that the classification performance of our model is higher for backbone‐based subselections compared to all‐atom representations. These findings indicate that dynamical differences in the agonist–antagonist behavior are primarily modulated by collective dynamics of TLR4/MD2 rather than specific ligand–residue interactions located at MD2. After analyzing the connectivity of the nodes along the simulations, MD2‐polar rim and surrounding regions play a different behavior upon the binding of agonist or antagonist glycolipids, indicating a key role in the modulation of TLR4 activity. This work shows how MD simulation analysis through GNNs provides a powerful tool to extract meaningful atomic information to identify functionally relevant binding features in protein‐ligand complexes.

## Graphical abstract

Graph neural networks (GNNs) have been applied to analyze molecular dynamics (MD) simulations of several complexes of Toll‐like receptor (TLR4) with glycolipids with agonist and antagonist activity. Dynamical differences in the complexes with agonists or antagonists have been found indicating that collective dynamics of the protein can modulate the agonist–antagonist behavior.

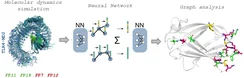

## 1 Introduction

Toll‐like receptors (TLRs) are a group of proteins of the innate immune system expressed in the cellular membrane or endosomes and are involved in the early detection of pathogen‐associated or damage‐associated molecular patterns (PAMPs and DAMPs, respectively) [1]. These molecular patterns are recognized by the extracellular domains of TLRs, which undergo conformational changes that trigger an appropriate immune response [2]. Among the different members of this family, TLR4 is one of the most extensively studied receptors and is associated with the recognition of lipopolysaccharide (LPS), a major component of the outer membrane of gram‐negative bacteria (Figure 1A) [3]. In the recognition of the LPS by TLR4, the myeloid differentiation factor 2 (MD2) plays a critical role [4]. This protein acts as a coreceptor of TLR4 and consists of a β‐cup‐like structure characterized by a hydrophobic cavity and a polar rim that allows the long hydrophobic fatty acid chains of the LPS to be buried inside the MD2 pocket, while the saccharide moiety remains exposed and can interact with the polar rim of MD2. This arrangement favors additional contacts between the polysaccharide portion of LPS and TLR4 itself (Figure 1A). Upon ligand binding, TLR4/MD2 dimerizes into the activated form (TLR4/MD2/LPS)_2,_ which triggers downstream cellular signaling pathways involved in the regulation of the innate immune responses [5].

**FIGURE 1:**
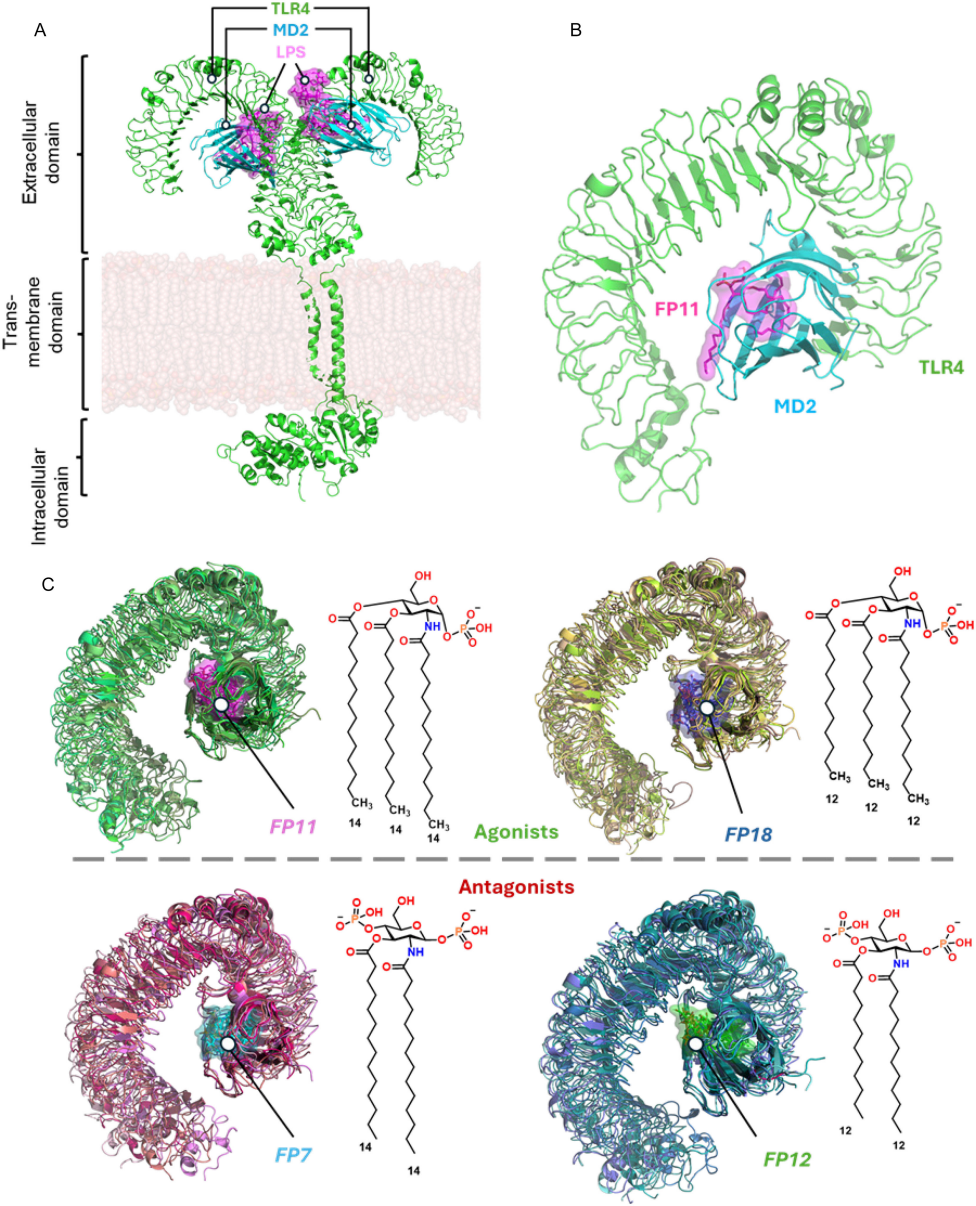
(A) 3D model of the full dimer TLR4/MD2/LPS, detailing the extracellular domain, the transmembrane domain, and the intracellular domain. (B) 3D structure of the TLR4/MD2/FP11 complex from reported computational studies [6]. TLR4 is shown in green, MD2 is shown in cyan, and glycolipid FP11 is shown in pink sticks and surface. (C) Superimposition of final snapshots from the MD simulations (5 replicas) of each TLR4/MD2 in complex with glycolipids FP11, FP18, FP7, and FP12. The 2D structures of the FP compounds are also shown.

The central role of TLR4 in innate immunity highlights its relevance as a therapeutic target for modulating immune signaling [1]. Activation of the TLR4 pathway has attracted considerable interest for the development of vaccine adjuvants, whereas inhibition of TLR4 activity represents a promising strategy for the treatment of inflammatory and autoimmune diseases, and cancer [7, 8, 9]. Accordingly, a wide variety of modulators targeting TLR4 has been reported to date, including peptides, lipid A analogs, monomeric glycolipids, and small‐molecule compounds [10, 11, 12, 13]. Several families of glycolipids (FP compounds) have been designed as antagonists and agonists of TLR4 [6, 14, 15], constituting an especially suitable model system for the design of TLR4 modulators (Figure 1B). These compounds consist of a single glucosamine core which is functionalized with two or three fatty acid chains at positions 2, 3, and 4 with variable chain lengths, and bearing one or two phosphate groups at position 1 and 4 (Figure 1C). Structure‐activity relationship studies revealed that compounds featuring a single phosphate group and a tri‐acyl substitution pattern showed agonist activity, whereas derivatives containing two phosphate groups and a di‐acyl substitution pattern exerted antagonist activity. Computational studies carried out by us on the binding mode of these glycolipids into TLR4/MD2 showed that both agonist and antagonist ligands bind inside the MD2 cavity [6, 15] (Figure 1B) in agreement with available X‐ray crystallographic structures of other TLR4 ligands. In these complexes, the polar saccharide headgroup interacts with the rim of MD2 through phosphate‐ and OH‐mediated contacts, whereas the fatty acid chains are accommodated within the MD2 hydrophobic pocket establishing hydrophobic interactions with aliphatic and aromatic residues of MD2.

Interestingly, despite the high structural similarity of these compounds and their conserved binding mode inside the MD2 pocket, subtle differences in acylation and phosphorylation patterns result in opposite functional responses. These findings highlight how minor chemical modifications in glycolipid derivatives can translate into distinct agonistic or antagonistic activities at the TLR4/MD2 complex. Although our combined docking and molecular dynamics (MD) simulations captured these subtle variations, the high dimensionality of the MD data could still prevent from finding deeper insights, given the difficulty of the extraction of functionally relevant information from large, high‐dimensional trajectories using predefined descriptors alone. In this context, graph neural networks (GNNs) have recently emerged as powerful approaches for analyzing MD simulations and for “decoding” the information contained in the protein–ligand interactions and in the conformational dynamics [16, 17].

GNNs can enrich the classical analysis of MD simulations, helping to process the large amount of data contained in such calculations, based on their ability to integrate structural, chemical, and temporal relationships between residues and ligands [18]. By learning directly from molecular topology, three‐dimensional geometry, and time‐dependent interactions, GNNs can extract relevant features from complex MD trajectories without relying exclusively on predefined descriptors (Figure 2). The molecular systems can be easily represented as graphs, where atoms are treated as nodes and their spatial relationships with surrounding atoms are encoded as edges. Once the 3D information is transformed into these graphs, GNNs can analyze these graph representations along different frames of the simulation, without requiring prior simplification of the system or the definition of specific variables. Also, GNN models can infer both local and nonlocal interactions through message passing between connected nodes. This ability makes these methods ideal for the study of flexible systems, such as protein–ligand complexes, where the biological activity often relies on global conformational changes rather than particular interactions with selected amino acids. GNNs may therefore complement conventional MD analysis by helping to identify molecular determinants of ligand recognition and functional responses such as agonism or antagonism. However, the application of these GNNs to the analysis of protein MD simulations is still a growing field rather than a well‐established method. These techniques can be used to identify relevant contributions at atomic‐ or residue‐level by mapping back the learned information to the molecular structure, linking the analysis to the physical and biological interpretation [18].

**FIGURE 2:**
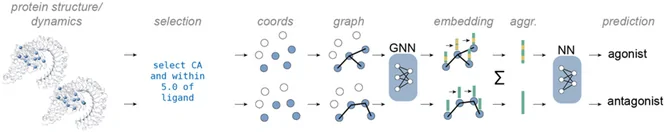
High‐level description of the prediction framework employed in this work. The machine‐learning task is to distinguish which protein‐structure dynamics correspond to agonists or to antagonists; for that, atoms of the proteins can be selected. Those selections can lead to sets of coordinates, which are transformed into graphs by considering fixed distance radius. The graphs are introduced into graph neural networks, which generate an embedding (vector, colored in green and yellow to indicate numerical differences). These embeddings are aggregated using a simple sum. The final outcome is analyzed by a feed‐forward neural network to classify the dynamics as agonists or antagonists. This framework enables us to test the role of some selections in the interaction with the ligand.

In the present work, we apply a graph convolutional neural network (GCNN) approach to analyze MD simulations of TLR4/MD2 complexes bound to structurally similar glycolipid ligands with either agonist or antagonist activity. By combining all‐atom MD simulations with a GNN approach, our aim is to identify the relevant regions that play a key role in the dynamical differences between agonist and antagonist states of TLR4/MD2, providing insights into how small changes in the chemical structure of the ligands translate into distinct biological responses. More generally, approaches capable of identifying the structural and dynamical features that govern the agonist/antagonist balance of receptor systems are of broad interest, as they may help to rationalize functional outcomes and guide the design of new modulators across different signaling pathways.

## 2 Results and Discussion

### 2.1 Data Set Acquisition: MD Simulations

Five replicas of MD simulations (150 ns each) of the TLR4/MD2 complex with the four glycolipids FP7, FP11, FP12, and FP18 were performed (Figure 1C). Starting geometries and docked poses were obtained from previous work where the glycolipids are inserted inside the MD2 pocket and the polar OH and phosphate groups establish electrostatic interactions and H‐bonds with the polar residues at the MD2 rim [6, 15]. In all cases, the ligands maintained the interactions inside MD2 along the simulations (Figure S2), and the receptor structure remained stable (Figure S2), keeping the previously reported binding mode.

### 2.2 GCNN

A GCNN was trained to distinguish graphs generated from subselections of trajectories including agonists or antagonists to determine the relevance of those subselections in the establishment of interactions of the receptor with agonist and antagonist glycolipids. Each frame extracted from the MD trajectories was treated as a snapshot of atomic spatial configurations that spanned the residue and atom types of the selected atoms. Graph‐convolutional neural networks were implemented using the PyTorch and PyTorch Geometric libraries following a split architecture that processed in parallel both residue embeddings and atom‐type embeddings. In the final layers of the model, attentional aggregation is employed to pool the information of the individual nodes, resulting in vectors which are merged and introduced in a linear layer to generate the final binary prediction (agonist vs. antagonist) (Figure 2).

Atom types and residues were mapped to residue‐specific and atom‐type‐specific embeddings. Graphs were generated from the atomic configurations using a distance‐based cutoff. To select appropriate cutoff values, a range of distances was systematically evaluated by analyzing the distribution of neighbors per node for each atomic representation, that is for backbone or all‐atom subselections (Figure S1). This analysis allowed us to identify values that provide a connected but nonsaturated graph, avoiding both sparse networks with insufficient interactions and overly dense graphs that may obscure relevant structural information. Based on this criterion, a cutoff of 5 Å was selected for backbone representations, in agreement with previous studies [19], while a cutoff of 4 Å was used for all‐atom representations.

### 2.3 Classification of Molecular Graphs Along Simulations

We aimed to obtain a GCNN model that captures the most important TLR4 receptor regions to distinguish agonist and antagonist behavior upon glycolipid binding along the dynamics simulations. To do so, we determined different atomic subselections to represent different structural and dynamical information, ranging from local ligand–residue interactions to collective conformational changes of the receptor. By comparing the accuracy of the model for each subselection, we can infer which regions of the receptor play a more relevant role in the dynamical shifting between agonist and antagonist behavior.

#### 2.3.1 Model Validation

We first optimized our model over three key variables: dataset size, frame splitting strategy, and atomic subselection. As our main goal is not the classification of dynamics but the detection of receptor regions involved in the distinction between agonists/antagonists, we adjusted the validation procedure to focus on the biological interpretability rather than maximizing the performance of classification on new data. As an initial protocol, we included 20 trajectories of 150 ns each and an atomic subselection of the backbone of MD2, which we hypothesized to undergo the most relevant conformational rearrangements associated with functional switching. We compared the use of the full simulation time (150 ns) with a reduced dataset comprising only the second half of the trajectories (76–150 ns), corresponding to a time window in which the systems have already evolved beyond the initial equilibration. When the full trajectories were employed, the model exhibited no discriminative power (accuracy 50%), indicating a high level of overfitting due to simulation run‐specific features. In contrast, when we analyzed the late‐stage dynamics, we found a marked improvement in classification performance (accuracy 92%; Table 1). These results indicate that the dynamical differences between agonist and antagonist systems become more pronounced at later stages of the simulations.

**TABLE 1:** Performance of the GCNN model under different training and validation setups.

Id	Employed time, ns	**Atomic subselection** **a**	Validation method	Accuracy, %
1	150	MD2 backbone	Random split	50
2	76–150	MD2 backbone	Random split	92
3	76–150	MD2 backbone	Manual split	51
4	76–150	TLR4 and MD2 backbone	Random split	97
5	76–150	MD2 polar rim	Random split	85
6	76–150	MD2 active	Random split	81

a The atomic selection corresponds to atoms named C, CA, N, and O for backbone (specified), or all‐atom for entries 5 and 6.

After this, we evaluated two different strategies to split simulation frames into training and test sets: in the first one, contiguous blocks of 100 frames corresponding to 1 ns were randomly assigned to the training or test sets (random split); in the second strategy, whole trajectories were manually separated, using four out of five trajectories per complex for training and the remaining trajectory for testing (manual split). The random split strategy yielded high classification accuracy (92%), whereas the manual split resulted in almost random performance (51%; Table 1). This difference is consistent with the strong temporal correlation between frames within the same trajectory, leading to an overestimation in the classification performance when adjacent frames are split between training and test sets. As our main goal differs from building an agonist/antagonist classifier but focuses instead on the analysis of trajectories, the random split strategy was selected for further analysis in the identification of dynamical differences between agonist and antagonist states in the simulated trajectories.

#### 2.3.2 Atomic Subselections in TLR4/MD2

Once the analysis framework was established (random split and restricted time window of 76–150 ns), we evaluated the performance of the neural network using different atomic subselections of the receptor to determine which regions provided better discrimination between agonist and antagonist states, under the assumption that higher classification accuracy reflects a greater contribution of the corresponding region to the agonist/antagonist behavior of the TLR4/MD2/glycolipid complex. Four atomic subselections were considered (Figure 3; detailed residues are in Supporting Information): (i) the backbone of the whole receptor (TLR4 + MD2; atoms C, CA, N, and O), (ii) the backbone of the binding protein alone (MD2; atoms C, CA, N, and O), (iii) all atoms of the polar rim of MD2, previously reported to undergo conformational rearrangements upon ligand binding, and (iv) all atoms of residues located within 5 Å of the ligand.

**FIGURE 3:**
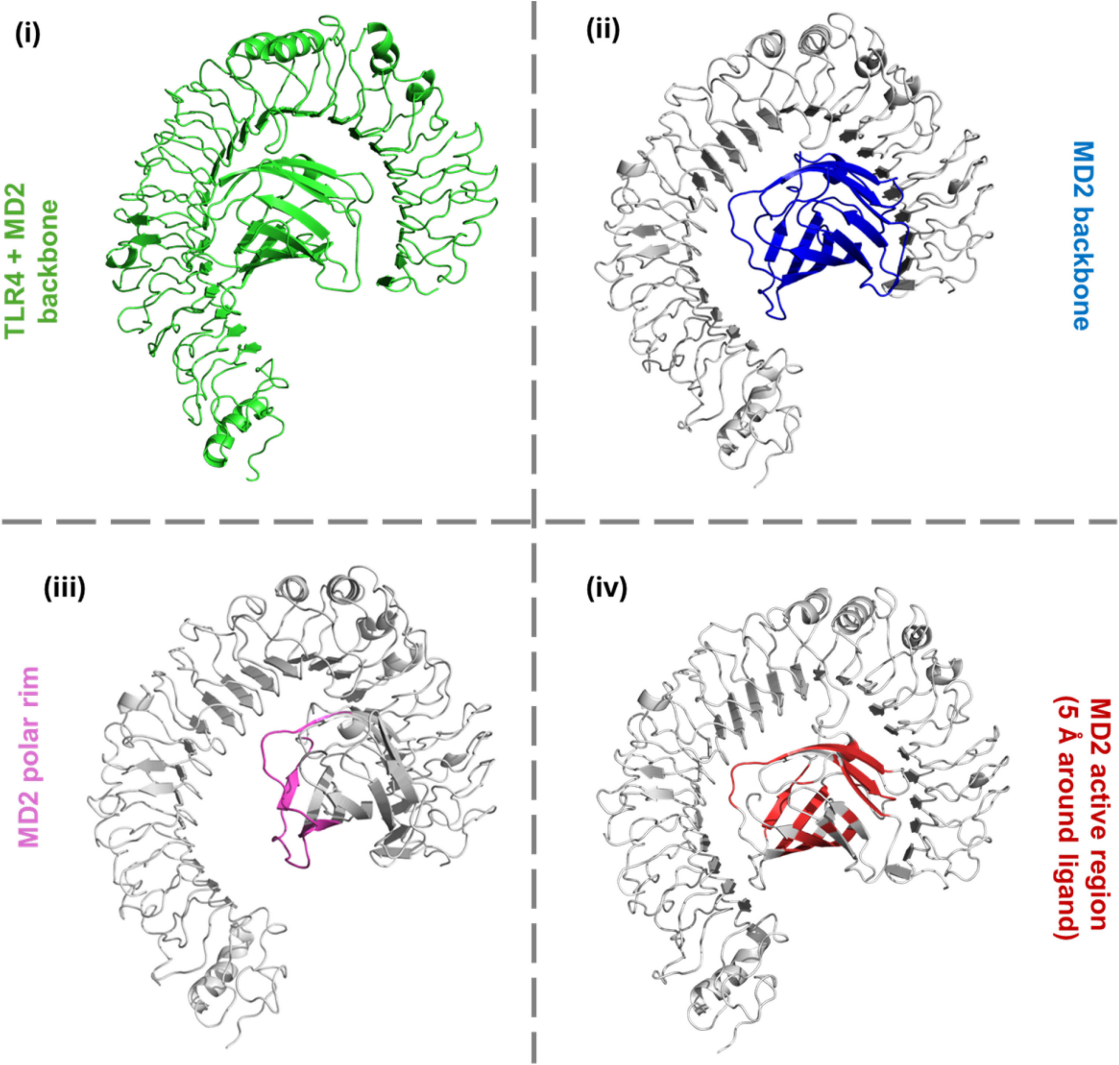
Detail in cartoon representation of the distinct atomic selections studied with the GCNN model in green (selection i), blue (selection ii), magenta (selection iii), and red (selection iv). Nonselected atoms of the rest of the simulated system are shown in gray.

i. First, we employed the backbone of the full TLR4/MD2 complex, obtaining the highest classification accuracy (97%, Table 1). These results indicate that the ability to distinguish between agonist and antagonist responses is strongly related to global or collective dynamics of the receptor. For this subselection, the model primarily highlights the relevance of long‐range structural conformational changes, which involve both TLR4 and MD2. Therefore, this high performance suggests that the dynamical differences between both states are not only related to the binding site but also to coordinated motions that involve the TLR4/MD2 complex as a whole.
ii. When restricting the analysis to the backbone of MD2 alone, the performance remained high (92%, Table 1), but slightly lower than the TLR4/MD2 subselection. This suggests that most of the relevant dynamical information is contained within MD2, which plays a critical role in ligand recognition. However, the decrease in accuracy indicates that contributions from TLR4 are in fact relevant, probably because the dynamical response relies on MD2 but is transmitted to TLR4, which seems to subtly modulate these conformational changes.
iii. On the other hand, when focusing on the all‐atom representation of the polar rim of MD2, the accuracy decreases (83%, Table 1), even when this region is known to undergo different conformational rearrangements upon the binding of an agonist or an antagonist. Thus, although the polar rim plays a relevant role in glycolipid recognition, the local side‐chain dynamics alone are not enough to fully distinguish between agonist and antagonist states. However, if we consider the small size of this subselection (defined as residues Ile80–Cys95 and Ser118–Lys132), this region still encodes a significant amount of information related to the system dynamics, suggesting that the polar rim contributes to the agonist–antagonist differentiation as part of a larger network of conformational changes rather than as an isolated structural element.
iv. Finally, when residues located within 5 Å around the ligand were considered, we found a lower classification performance (79%, Table 1), suggesting that direct ligand–residue interactions, despite being essential for binding, only provide a limited view of the global dynamics. In comparison with the results of the polar rim subselection (iii), this region is mainly related to the near environment of the ligand, lacking part of the structural context, which is required to fully understand the conformational changes between both states. These results support the idea that, while local interactions initiate the dynamic response, the relevant dynamical signal that allows the model to discriminate between agonist or antagonist states is more related to how these interactions are transmitted, first to MD2 and later to TLR4, finally leading to distinct global conformational behaviors.

Previous structural and computational studies of the TLR4/MD2/LPS complex support this interpretation. Crystallographic analyses of the agonist‐bound receptor have shown that ligand binding is followed by rearrangements in both MD2 and TLR4 ectodomains, including changes in receptor geometry that extend throughout the receptor, not only to the binding pocket [4]. Additionally, full‐length simulations also indicate that agonist binding stabilizes collective motions of the ectodomain while maintaining the characteristic configuration of the MD2 loop encompassing residues 123–129, acting as a conformational switch during activation [5]. Together, these observations suggest that the agonist–antagonist distinction is primarily associated with the propagation of dynamical changes through the TLR4/MD2 scaffold rather than with specific local ligand–residue interactions. This interpretation is consistent with the higher performance observed for backbone‐based representations, which appear to capture these collective conformational changes more effectively than localized all‐atom subselections.

### 2.4 Graph Analysis

To further interpret the dynamical differences captured by the model, we analyzed the changes in node connectivity throughout the simulations. In this context, these changes indicate differences in the frequency of residue–residue contacts, describing how the local environment of each residue changes upon ligand binding. Nodes were ranked according to the magnitude and sign of their connectivity changes, and the top and bottom 10% were selected. The top 10% of positively ranked nodes corresponded to connectivity changes associated with the agonist state, whereas the bottom 10% highlighted nodes linked to changes promoting the antagonist state. These nodes were subsequently mapped back to their corresponding atoms and residues, allowing us to identify, for each atomic subselection, the regions of the receptor that experienced the greatest dynamical differences upon binding of agonist or antagonist ligands (Figure 4A; detailed residues are in Supporting Information).

**FIGURE 4:**
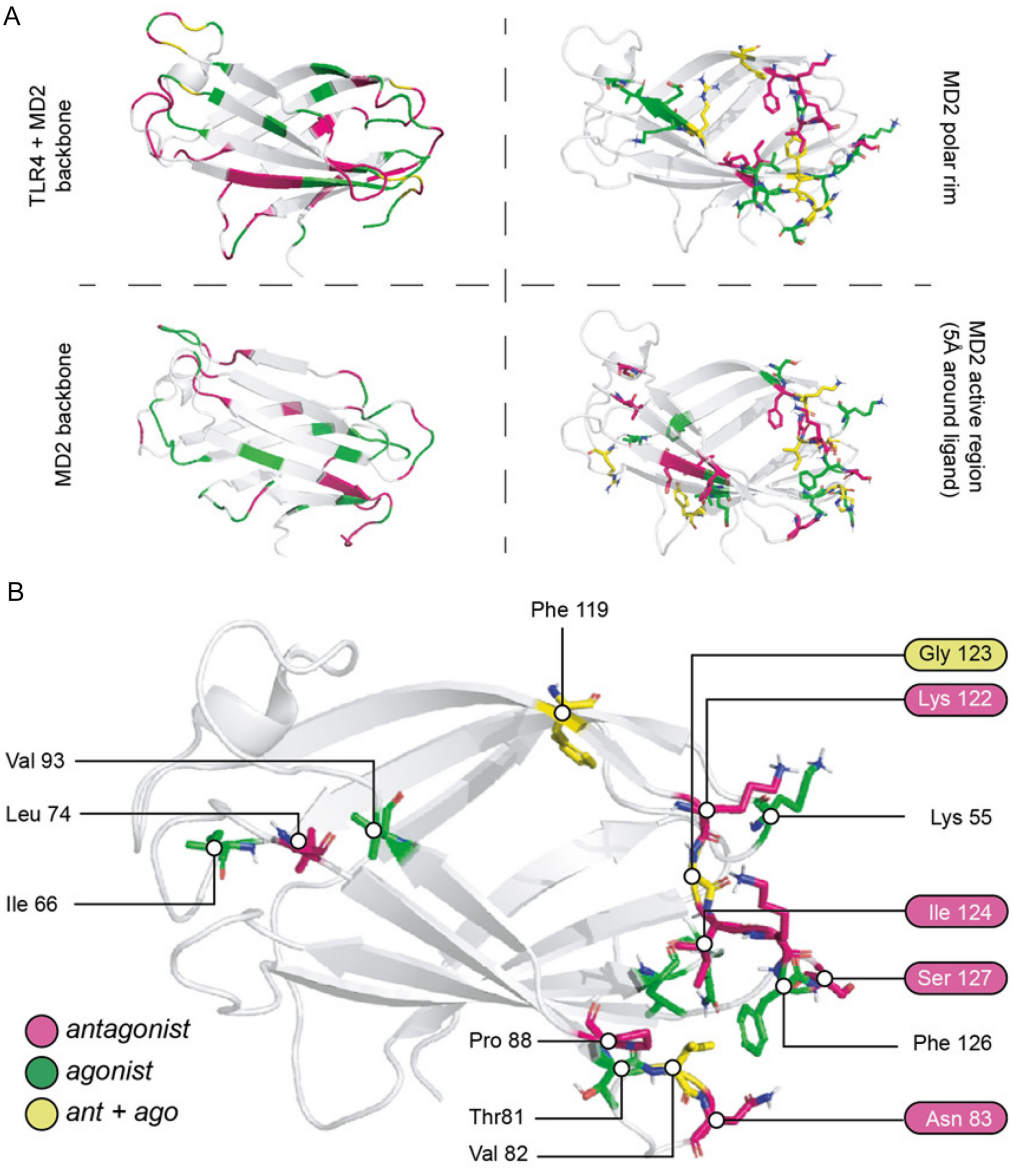
(A) Detail in cartoon representation of the amino acids of MD2 that contains nodes marked as relevant (top and bottom 10% ranked nodes) by the graph analysis for each atomic selection. Green amino acids are those related with the agonist response, pink residues are those related with the antagonist response and amino acids that contain nodes relevant to both the agonist and antagonist response are marked in yellow. (B) Detail of the amino acids of MD2 that contains nodes marked as relevant by the graph analysis in at least three out of four atomic selections. Those amino acids with a label in a box were marked as relevant by the graph analysis in all the atomic selections.

Our analysis showed that nodes that are part of MD2 exhibited the largest connectivity changes during the simulations. Given the central role of MD2 in ligand recognition, the difference between agonist and antagonist states seems to be related to changes in the interaction network of MD2 residues. However, when considering backbone‐based atomic subselections (TLR4/MD2 or MD2 alone), as well as the all‐atom subselection of residues surrounding the glycolipid, the top‐ and bottom‐10%‐ranked nodes mainly correspond to individual residues distributed all across the β‐cup. These results indicate that these changes in the interaction pattern are spread across different regions of the protein, suggesting that these local changes contribute to the global conformational changes observed upon ligand binding.

Additionally, our analysis revealed that several residues within the polar rim contained at least one relevant node across all graph analyses, independently of the atomic subselection considered (Figure 4B). This convergence indicates that, despite the global nature of the signal, certain regions of MD2 play a key role in the conformational differences between agonist and antagonist responses. A group of amino acids (Lys55, Ile66, Leu74, Thr81, Val82, Pro88, Val93, Phe119, and Phe126) was identified in at least three of the four subselections, whereas a smaller group (Asn83, Lys122, Gly123, Ile124, and Ser127) was consistently detected across all subselections (Figure 4B). These residues are mainly located in the polar rim of MD2, surrounding the β‐cup cavity, a structural element known to play a central role in the recognition of LPS and related glycolipids. In particular, per‐residue energy decomposition identified Pro88 as making favorable contributions at the activation‐associated TLR4/MD‐2 interface in LPS–bound complex [20]. Also, Lys122 and Ser127 have been identified as a crucial position at the MD2 rim for species‐specific endotoxin sensing with a stabilizing role for the antagonist orientation of lipid IVa in human TLR4/MD2 [21]. Gly123 and Ile124 follow Lys122 and belong to the key Phe126 loop, whose conformational state is coupled to ligand agonism/antagonism and TLR4 dimerization, acting as an on/off switch [5, 20, 22]. Additionally, mutations of some of these residues have been reported to worsen ligand binding. For example, mutation of Phe119 to Ala led to decrease of LPS binding, while mutation of Lys122 to Pro altered binding affinity likely due to the change in the local electrostatic potential [23, 24].

To further investigate the role of these residues, we computed residue–residue contact maps for all replicas of the four TLR/MD2 complexes with FP7, FP11, FP12, and FP18. Remarkably, the five identified residues (Asn83, Lys122, Gly123, Ile124, and Ser127) displayed highly similar contact patterns with both TLR4 and MD2 across all systems, independently of whether the bound ligand was an agonist or an antagonist (Figure S3). This reinforces the idea that the agonist or antagonist behavior does not arise from specific ligand‐dependent interactions at these positions but from subtle dynamical rearrangements that propagate through the MD2 β‐cup and into TLR4.

These loops combine structural flexibility with a direct involvement in glycolipid binding, allowing them to adapt to local changes while also transmitting these changes to the rest of the protein. Thus, residues that appear repeatedly across all graph analyses seem to participate in the spread of the dynamical signal induced by ligand binding, rather than simply contributing to ligand–residue interactions. Also, the residue–residue contact map shows that the residues consistently identified across all graph analyses do not exhibit ligand‐dependent changes in their local interaction network. Overall, these results highlight the polar rim of MD2 as a key structural region where local interactions, flexibility, and global conformational changes converge to modulate the functional shift between agonist and antagonist responses of the TLR4/MD2 complex. These results agree with the ligand‐receptor interactions identified from the reported simulations of all these glycolipids [6, 15] and mutational analysis [23].

## 3 Conclusion

In this work, we explore the dynamical basis of agonist or antagonist responses in TLR4/MD2/glycolipid complexes through a GCNN‐based analysis of the MD simulations. Our results show a more prominent role of the backbone‐based subselection in comparison with the more localized all‐atom subselections, which suggests that differences between both states are more related to changes in the interaction network across the receptor, rather than to particular ligand–residue contacts.

The integrative analysis of the graphs obtained from multiple atomic subselections reveals the importance of certain regions that may remain unnoticed if we analyze these graphs individually, in particular, the polar rim and the surrounding loops of MD2. Our results show that these regions display a differential behavior upon the binding of an agonist or antagonist glycolipid. At the same time, these loops seem to play a key role in the spread of dynamical changes from local interactions to global conformational changes of the TLR4/MD2 complex.

From a methodological point of view, these results show that deep‐learning algorithms, combined with subsequent graph‐based analysis, can be used to extract physically meaningful information from MD simulations. By mapping back graph‐based information to particular atoms and residues, this approach allows the identification of regions that undergo the most relevant dynamic changes upon the binding of distinct agonist or antagonist glycolipids, providing valuable insights for future optimization of glycolipids as TLR4 modulators. Additionally, this approach surfing across different atomic subselections could also be applied to other protein–ligand systems, particularly in cases where limited experimental or structural information is available. Extracting patterns from noisy data is one of the fundamental quests of computational structural biology, for which modern machine learning techniques represent a particularly promising tool.

## Funding

This work was supported by the Spanish Ministry of Science, Innovation, and Universities (PID2020‐113588RB‐I00, PID2023‐152271NB‐I00, and PID2021‐124671OB‐I00), and FEDER, UE (PID2021‐124671OB‐I00).

## Conflicts of Interest

The authors declare no conflicts of interest.

## Supporting information

MD simulation methodology. Graph convolutional neural network. Residues of each subselection. Top ranked residues after graph analysis. A “Guidance for the case study: choice of customized atomic subselection based on knowledge” is also provided in the Supporting Information. The starting frames from which molecular dynamics were obtained are available at the Zenodo repository at https://www.doi.org/10.5281/zenodo.22796892. The code employed to build, train, and test our models is available at https://github.com/brunocuevas/MLMD25.

## Data Availability

The data that support the findings of this study are openly available in Zenodo at https://www.doi.org/10.5281/zenodo.22796892.
