# Supplementary material for: Dynamic Determinants of the TLR4/MD2/Glycolipid Binding Unveiled Through Graph Neural Networks
Source: Chembiochem. 2026 Nov 14;27(22):e70581. doi: 10.1002/cbic.70581 (PMC13769024; doi:10.1002/cbic.70581)
Supplement: Supplementary file 1 [file CBIC-27-e70581-s001.pdf]

Supporting Information

Dynamic determinants of the TLR4/MD2/glycolipid binding unveiled through graph neural networks

Olmo Martín-Cámara,[a] Álvaro Arrayás-Ruiz,[a] Bruno Cuevas-Zuviría,*[b] Sonsoles Martín-Santamaría*[a]

[a] O. Martín Cámara, A. Arrayás-Ruiz, S. Martín-Santamaría*
Centro de Investigaciones Biológicas Margarita Salas, CSIC.
C/ Ramiro de Maeztu, 9. 28040-Madrid, Spain.
E-mail: smsantamaria@cib.csic.es

[b] B. Cuevas-Zuviría*
Centro de Biotecnología y Genómica de Plantas, Universidad Politécnica de Madrid, UPM-INIA-CSIC
Campus de Montegancedo, Pozuelo de Alarcón, 28223-Madrid, Spain
E-mail: bruno.czuviria@upm.es

MD simulations methodology

As starting 3D geometries of the two complexes with antagonists FP7 and FP12, we employed the reported models of monomer TLR4/MD2 in the antagonist conformation, where FP7 and FP12 were docked.[1] As starting 3D geometries with agonists FP11 and FP18, we superimposed into this monomer TLR4/MD2 complex the 3D docked poses of FP11 and FP18 from reported 3D complexes with the $(TLR4/MD2)_2$ dimer.[2] In this way, we obtained four different complexes sharing the 3D structure of monomer TLR4/MD2, but differing in the bound pose of the four different glycolipids.

Five replicates of MD simulations of 150 ns were performed for each complex (TLR4/MD2/FP7, FP11, FP12 and FP18) employing Amber22 (ff14SB).[3] For these simulations an octogonal simulation box with a distance of 10 Å between any atom and the closer edge was employed, and then it was solvated using the TIP3P model and Na^+ and Cl^- were added to neutralize the charges of the system. After this, we performed a five steps minimization which consisted of an initial combination of 1000 steps of steepest descent minimization followed by a 7000 steps of conjugate gradient minimization, applying a restraint of 100 kcal mol^{-1} $Å^{-2}$ harmonic potential to the complex. The minimization continued through an iterative circle of 600 steps of conjugate gradient algorithm applying a less restrictive restraint each iteration (10, 5, 2.5 and 0 kcal mol^{-1} $Å^{-2}$ harmonic potential). The next step was an equilibration which was performed in three different steps: an initial heating from 0 to 100 K using canonical ensemble with the Langevin thermostat (NVT) applying an harmonic restrain of 10 kcal mol^{-1} $Å^{-2}$ to the complex; a second heating step from 100 to 300 K using an isothermal-isobaric ensemble (NPT), still maintaining the same restrain to the complex; and a final short simulation of 100 ps. During the simulation a time step of 2 fs and the Langevin thermostat were employed.

Graph Convolutional Neural Network

The neural network was trained using the Adam optimizer with an initial learning rate of 0.001 and halving learning-rate schedules. A L2-norm regularization with value 0.0005 was applied to constraint the capacity of the network and avoid overfitting. Some training hyper-parameters such as training epoch

size were optimized through a grid search, while the selection employed to subselect each dataset is the principal factor that will be considered.

To quantify differences between agonist and antagonist states, the corresponding weighted graphs were compared by computing the difference in edge weights between both systems. These edge-based differences were then projected onto nodes by summing the contributions of all edges connected to each node, providing a residue-level measure of connectivity change.

Residues of each subselection

The distinct atomic selections studied with the GCNN model are (see also Figure 2):

TLR4-MD2 backbone (atom name C, CA, O and N). TLR4: from Glu27 to Cys627; MD2: from Gln19 to Asn158.

MD2 backbone (atom name C, CA, O and N). From Gln19 to Asn158.

MD2 polar rim all-atom. From Ile80 to Cys95 and from Ser118 to Lys132.

Selected residues at 5Å around ligand. MD2: Val25, Ile32, Pro43, Ile44, Ser45, Ile46, Asn47, Val48, Ile52, Leu61, His52, Ile53, Phe64, Tyr65, Ile66, Leu71, Leu74, Tyr75, Phe76, Asn77, Leu78, Tyr79, Ile80, Arg90, Lys91, Glu92, Val93, Ile94, Phe104, Val113, Asn114, Thr115, Thr116, Ile117, Ser118, Phe119, Ser120, Phe121, Lys122, Gly123, Ile124, Lys125, Lys130, Tyr131, Lys132, Cys133, Val134, Val135, Leu146, Phe147, Cys148, Leu149, Glu150, Phe151, Val152, Ile153.

Top ranked residues after graph analysis

For each subselection, MD2 residues that contained nodes classified at the top and bottom 10%, in terms of changes in their connectivity, were extracted. Those residues were then classified into agonist, antagonist or relevant but not determinant if the contained nodes were in top, bottom or the residue contained a mixture of top and bottom nodes, respectively (see also Figure 3). The selected residues are:

For MD2-backbone selection

Agonist. Val24, Ala30, Ser31, Cys37, Lys39, Gln41, Pro43, Leu54, Lys55, Lys58, Tyr65, Ile66, Pro67, Arg68, Arg69, Asp70, Thr81, Val82, Asn83, Leu87, Arg96, Gly97, Asp99, Asp100, Gly123, Ile124, Ser127, Lys128, Lys132, Gly140, Cys178, Leu149, Gln156.

Antagonist. Ser28, Asp29, Asp38, Ile52, Gln73, Leu74, Pro88, Val93, Ile94, Ser98, Asp101, Lys109, Phe119, Lys122, Phe126, Gly129, Ile138, Ser139, Val152, Ile153, Leu154, His155, Pro157, Asn158, Ser159, Asn160.

For TLR4+MD2-backbone selection

Apart from TLR4 residues (data not shown), for MD2 top ranked residues are:

Agonist: Gln21, Ser27, Ser28, Asp29, Asp38, Ile52, Glu53, Leu60, Gln73, Tyr75, Leu78, Pro88, Val93, Ile94, Ser98, Asp101, Phe119, Ser120, Lys122, Lys125, Phe126, Glu129, Tyr131, Ile138, Ser139, Phe151, Val152, Ile153, Leu154, Asn158, Ser159, Asn160.

Antagonist: Trp23, Val24, Ala30, Ser29, Tyr36, Cys37, Lys39, Gln41, Pro43, Val48, Pro50, Leu54, Lys55, Lys58, Tyr65, Ile66, Pro67, Arg69, Asp70, Leu71, Lys72, Thr81, Val82, Asn83, Thr84, Met85, Leu87, Glu97, Asp99, Gly110, Gly123, Ile124, Ser127, Lys128, Lys132, Gly140, Ser141, Cys148, Leu149, Glu150, Gln156.

Relevant but not determinant: Gly56, Leu74, Arg96, Asp100, Lys130, His155, Pro157.

For MD2-polar rim selection

Agonist: Thr81, Tyr84, Asn86, Leu87, Lys91, Glu92, Val93, Cys95, Gly123, Lys128, Gly129, Lys130.

Antagonist: Ile80, Pro88, Phe121, Lys122, Ile124, Lys125, Ser127.

Relevant but not determinant: Val82, Asn83, Arg90, Phe119, Tyr131.

For MD2-selected residues

Agonist: Lys55, Phe64, Ile66, Ser120, Phe126, Val134, Glu150, Leu154, Gln156.

Antagonist: Leu74, Tyr79, Asn83, Cys95, Phe121, Gly123, Ser127, Val135, Glu136.

Relevant but not determinant: Ser27, Tyr36, Arg68, Lys122, Ile125, His155.

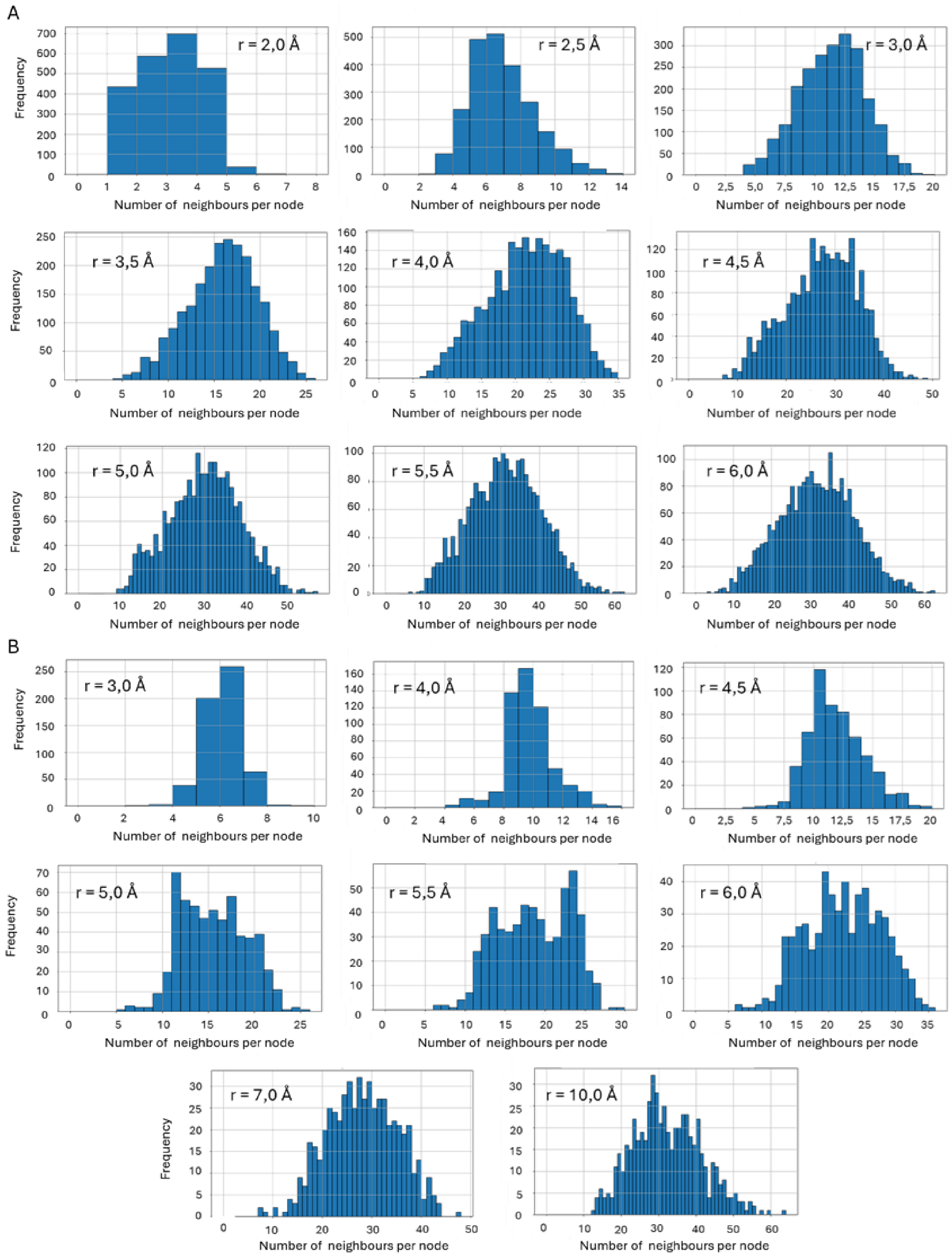

Figure S1. Distribution of the number of neighbors per node as a function of the distance cutoff used to construct the graphs for (A) backbone-based representation, and (B) all-atom representation, where nodes correspond to individual atoms. The distributions illustrate how different cutoff radii affect graph connectivity. In backbone-based graphs larger cutoff values were required to ensure sufficient connectivity. In contrast, all-atom representations, characterized by a higher node density, required smaller cutoffs to avoid overly dense and saturated graphs.

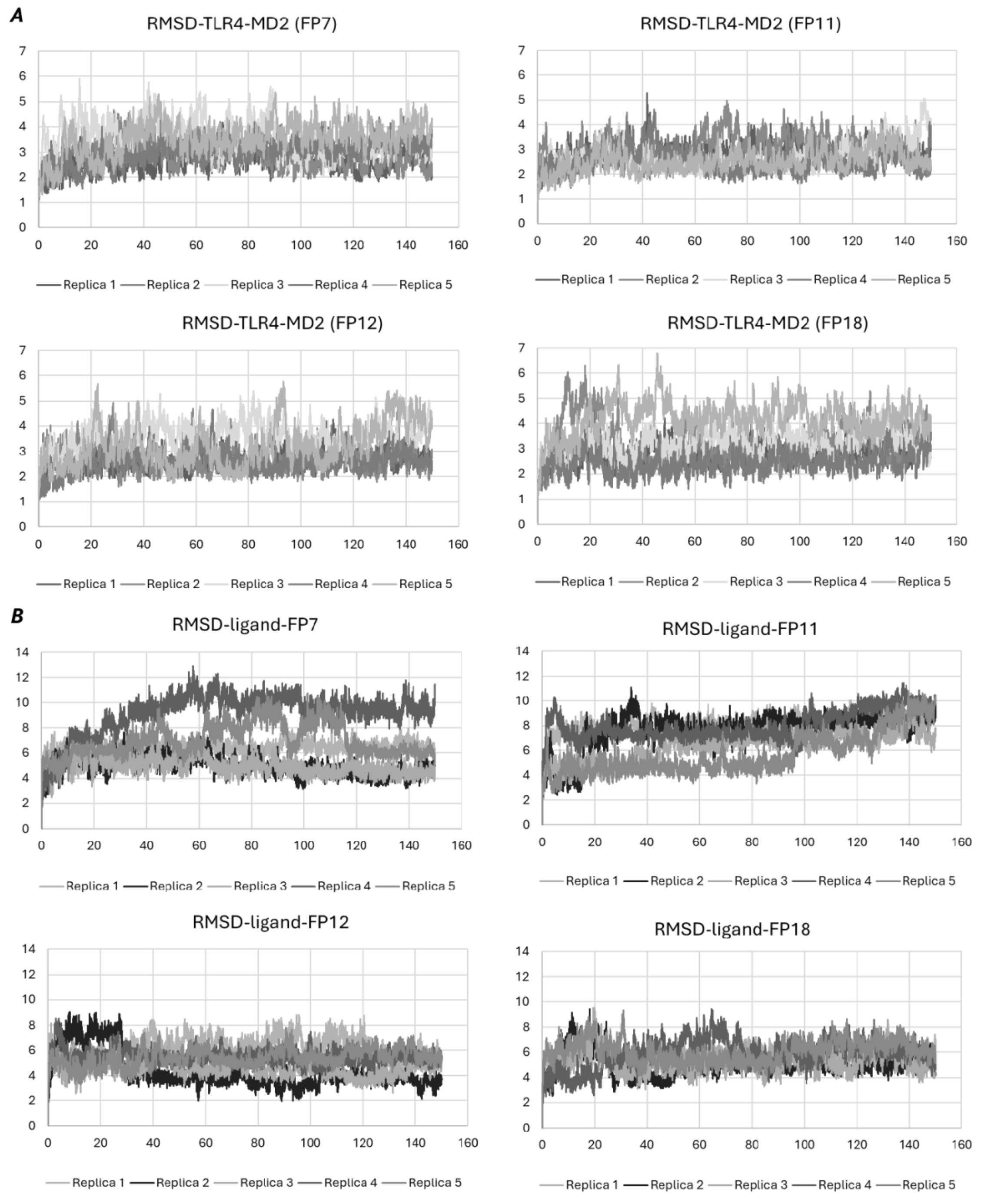

Figure S2. RMSDs of the TLR4/MD2/glycolipid complexes along the 150 ns of the simulation (5 replicas). A) RMSD for TLR4/MD2 (α-carbon); B) RMSD for the ligands FP7, FP11, FP12 and FP18 (heavy atoms).

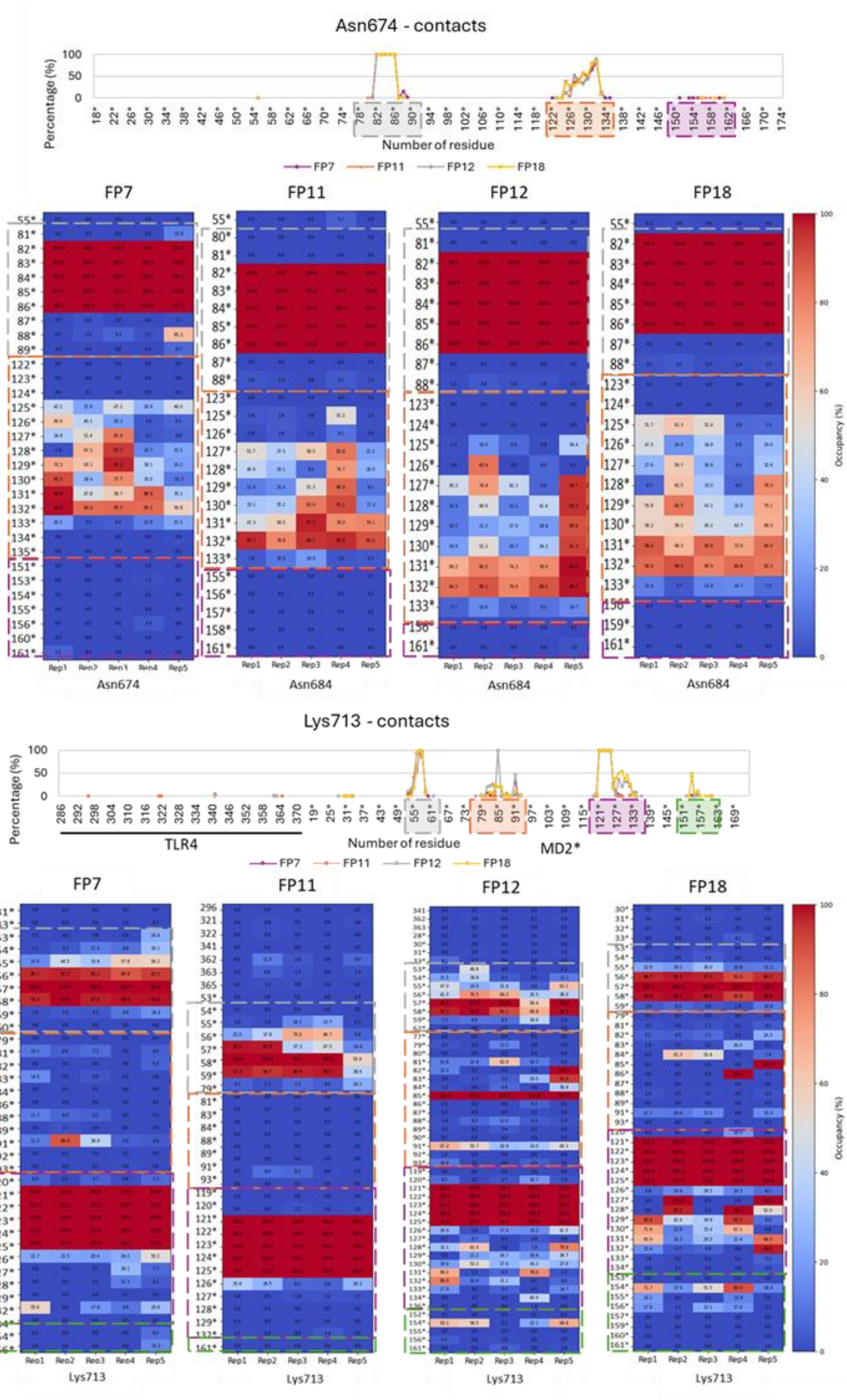

Figure S3. Contact maps of the selected residues with other residues of the receptor (TLR4 and MD2) calculated for each complex with ligands FP7, FP11, FP12 and FP18. The graphs on top represent the average occupancy values across the five replicas for each complex. Colored rectangles along the X axis highlight high-occupancy regions, which are also indicated in the tables below.

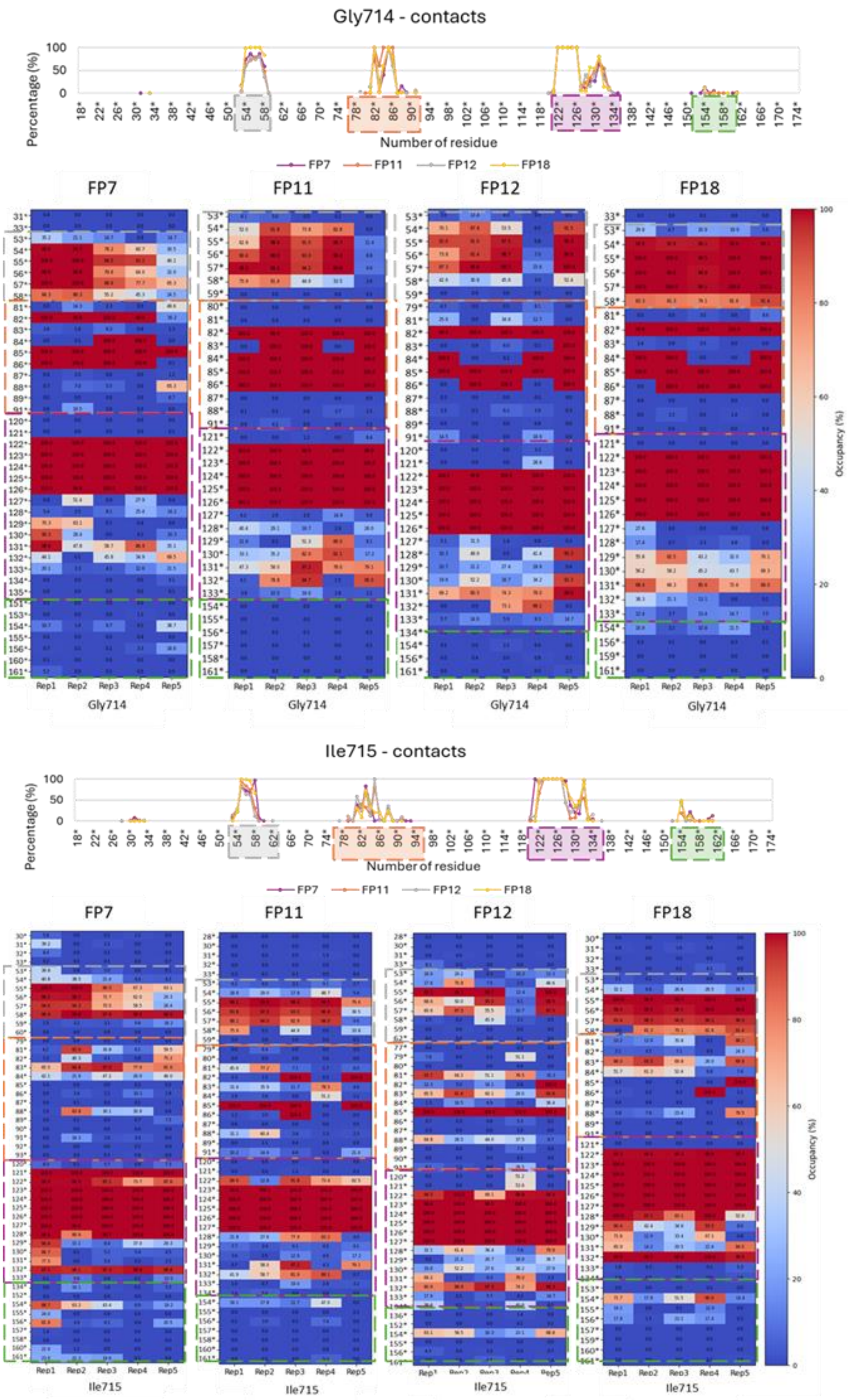

Figure S3. *Cont.* Contact maps of the selected residues with other residues of the receptor (TLR4 and MD2) calculated for each complex with ligands FP7, FP11, FP12 and FP18. The graphs on top represent the average occupancy values across the five replicas for each complex. Colored rectangles along the X axis highlight high-occupancy regions, which are also indicated in the tables below.

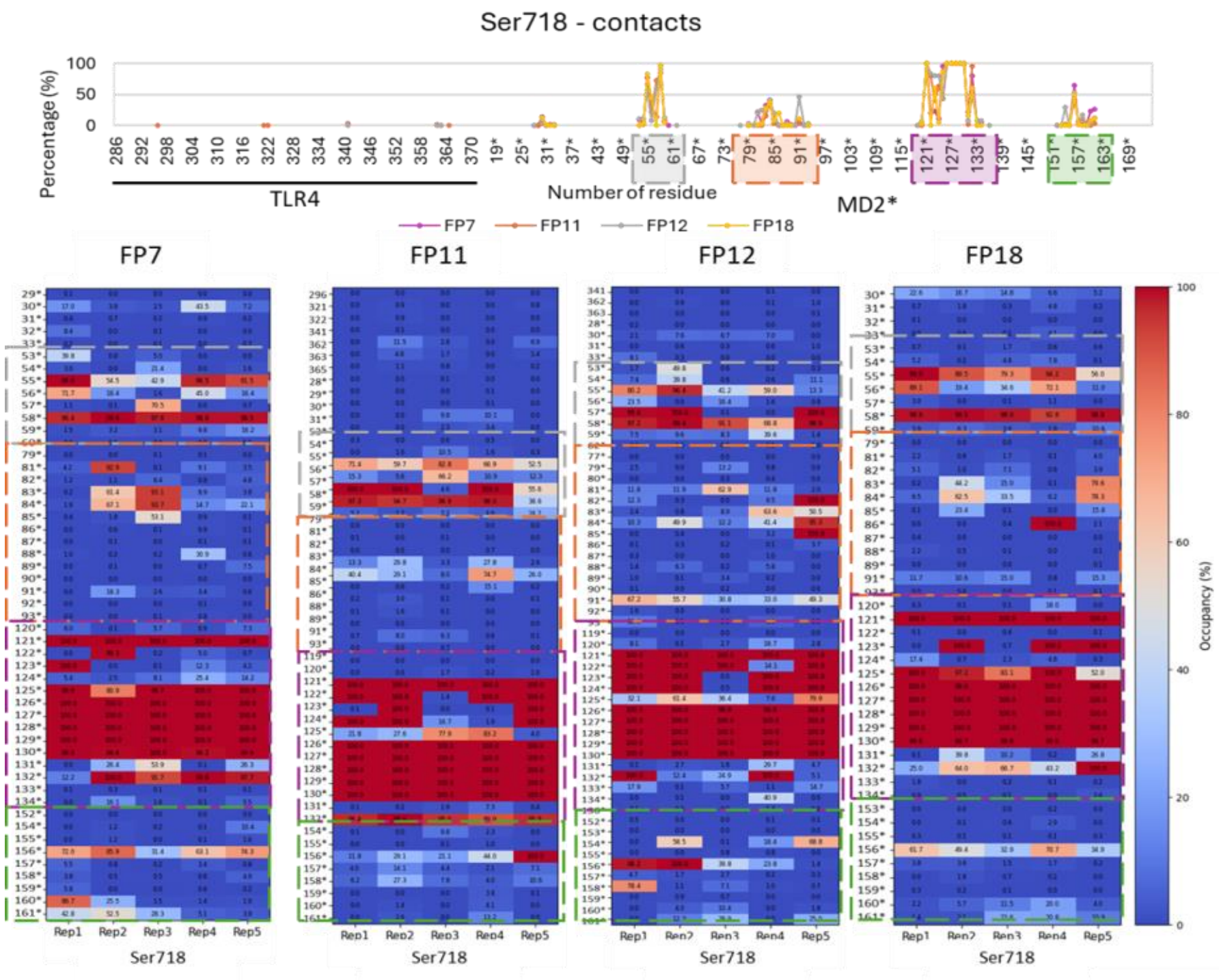

Figure S3. *Cont.* Contact maps of the selected residues with other residues of the receptor (TLR4 and MD2) calculated for each complex with ligands FP7, FP11, FP12 and FP18. The graphs on top represent the average occupancy values across the five replicas for each complex. Colored rectangles along the X axis highlight high-occupancy regions, which are also indicated in the tables below.

Supplementary References

[1] F. A. Facchini, L. Zaffaroni, A. Minotti, S. Rapisarda, V. Calabrese, M. Forcella, P. Fusi, C. Airoldi, C. Ciaramelli, J. M. Billod, A. B. Schromm, H. Braun, C. Palmer, R. Beyaert, F. Lapenta, R. Jerala, G. Pirianov, S. Martin-Santamaria, F. Peri, "Structure-activity relationship in monosaccharide-based toll-like receptor 4 (TLR4) antagonists" *J. Med. Chem.* **61**, (2018): 2895.

[2] F. A. Facchini, A. Minotti, A. Luraghi, A. Romerio, N. Gotri, A. Matamoros-Recio, A. Iannucci, C. Palmer, G. Wang, R. Ingram, S. Martin-Santamaria, G. Pirianov, M. De Andrea, M. A. Valvano, F. Peri, "Synthetic Glycolipids as Molecular Vaccine Adjuvants: Mechanism of Action in Human Cells and in Vivo Activity" *J. Med. Chem.* **64**, (2021): 12261.

[3] D. A. Case, D. S. Cerutti, V. W. D. Cruzeiro, T. A. Darden, R. E. Duke, M. Ghazimirsaeed, G. M. Giambaşu, T. J. Giese, A. W. Götz, J. A. Harris, K. Kasavajhala, T. S. Lee, Z. Li, C. Lin, J. Liu, Y. Miao, R. Salomon-Ferrrer, J. Shen, R. Snyder, J. Swails, R. C. Walker, J. Wang, X. Wu, J. Zeng, T. E. Cheatham, D. R. Roe, A. Roitberg, C. Simmerling, D. M. York, M. C. Nagan, K. M. Merz, "Recent Developments in Amber Biomolecular Simulations" *J. Chem. Inf. Model.* **65**, (2025): 7835.

Guidance for the case study:

choice of customized atomic subselection based on knowledge

Dynamic determinants of the TLR4/MD2/glycolipid binding unveiled through graph neural networks

Olmo Martín-Cámara,[a] Álvaro Arrayás-Ruiz,[a] Bruno Cuevas-Zuviría,*[b] Sonsoles Martín-Santamaría*[a]

[a] O. Martín Cámara, A. Arrayás-Ruiz, S. Martín-Santamaría*
Centro de Investigaciones Biológicas Margarita Salas, CSIC.
C/ Ramiro de Maeztu, 9. 28040-Madrid, Spain.
E-mail: smsantamaria@cib.csic.es

[b] B. Cuevas-Zuviría*
Centro de Biotecnología y Genómica de Plantas, Universidad Politécnica de Madrid, UPM-INIA-CSIC
Campus de Montegancedo, Pozuelo de Alarcón, 28223-Madrid, Spain
E-mail: bruno.czuviria@upm.es

This document summarizes the workflow developed for choosing atomic subselections when training Graph Convolutional Neural Networks (GCNNs) on Molecular Dynamics (MD) trajectories of protein-ligand complexes. It is meant as a practical guide for researchers facing a similar problem on a different system. Full derivations, results, and discussion are included in the full article.

The workflow was developed and validated on the TLR4/MD2 receptor system, Toll-like receptor 4 and its co-receptor MD2, a complex that recognizes both agonist and antagonist ligands in the same binding pocket. Applied examples from that case study are shown throughout *in italics*, so it can be seen the general recommendations put into practice.

The five phases move from general context to a specific validated answer, with the process looping back on itself because a subselection from Phase 2 that is only partially informative in Phase 3 is refined and tested again until ready to move to Phase 4 (Figure 1).

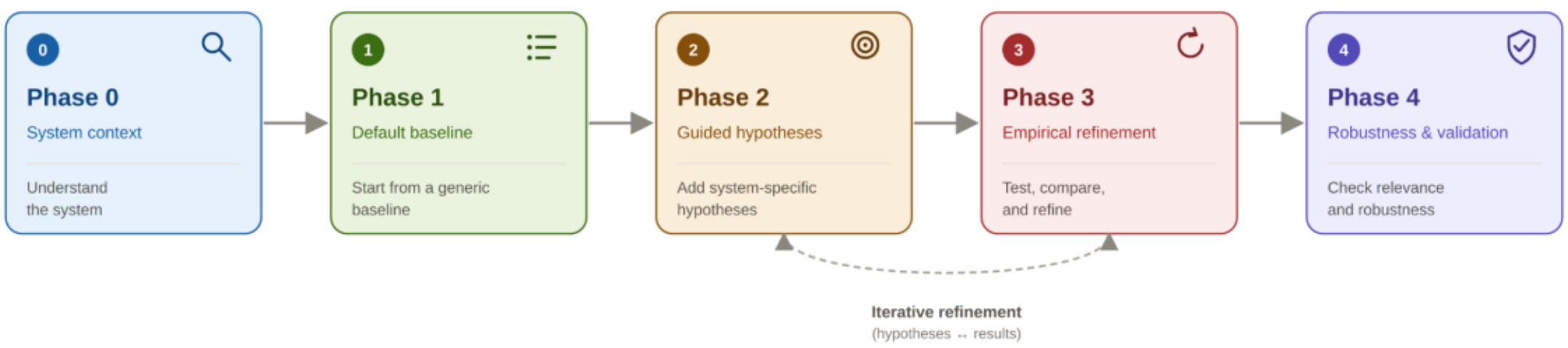

Figure 1. Visual Workflow for Atomic Subselection Design and Validation of Protein-Ligand Systems

Introduction

The choice of atomic subselection is not arbitrary, and it does not need to be fixed for a single protein-ligand system either. We approach this choice as a hybrid, iterative process, in which structural knowledge and model performance inform one another throughout the analysis, instead of treating it as a purely data-driven parameter or a purely knowledge-based one.

We recommend a workflow that:

- Begins from a small set of default, structurally-agnostic subselections (e.g. backbone or active regions).
- Progressively refines these subselections using prior knowledge of the system under study, such as binding site topology, known activation motifs, domain architecture and allosteric hinges.
- Uses the GCNN's own predictive performance and its attribution signal as a diagnostic criterion to determine which refinements are biologically meaningful and worth pursuing further.

Biological interpretation and model performance are not two separate stages of the analysis, they are tightly coupled at every step. Prior knowledge is what makes a subselection worth testing in the first place, while model performance is what tells you whether that piece of knowledge actually explains the discriminative signal in the data, or whether it was a reasonable-sounding guess that the dynamics don't support.

Neither is sufficient alone: knowledge without empirical testing risks confirmation bias: you find what you expected to find because you only looked there. Model performance without biological grounding risks an uninterpretable black box that discriminates well for reasons that cannot be mapped back to mechanism.

The workflow below exists to keep these two in continuous dialogue.

Phase 0.- System characterization

Before any atomic subselection is defined, the researcher should compile the structural and functional knowledge already available for the system of interest. This includes:

- The location and topology of the binding site(s).
- The domain architecture of the receptor.
- Any existing structure-activity relationship (SAR) data distinguishing active from inactive or agonist from antagonist ligands.
- Any prior structural or biochemical evidence pointing to conformationally relevant regions: hinge regions, allosteric interfaces, loops implicated in activation.

This phase does not yet involve selecting atoms, as its purpose is to establish the minimum floor of biological context needed to justify departing from a fully generic subselection strategy in later phases. In the absence of such knowledge, the workflow can still proceed, but refinement in Phase 2 will necessarily rely more heavily on the empirical signal obtained in Phase 3.

For TLR4/MD2, this included the MD2 hydrophobic pocket, the polar rim known to mediate polar contacts with the ligand headgroup, and prior docking/MD evidence that both agonists and antagonists occupy the same MD2 cavity despite opposite functional outcomes.

Phase 1.- Default baseline subselection

Independent of the specific system under study, we recommend establishing a baseline using a small set of subselections that require no specialized prior knowledge and can be defined directly from the receptor-ligand structure:

- Backbone atoms of the receptor, to capture global fold dynamics.
- All heavy atoms of the ligand.
- Residues within a fixed distance cutoff of the ligand (a contact-shell subselection).

Where the binding mode involves a solvent-exposed interface, a polar or charged-rim subselection can also be included at this stage as a further default option. This baseline set serves two purposes: it provides a first estimate of where discriminative signals are located in the system, and it establishes the reference performance against which every later knowledge-driven refinements must be compared.

In our study, the MD2 backbone served this role as the initial hypothesis, chosen because collective backbone motion was expected, a priori, to be the most likely carrier of a functional signal. The polar rim and the ligand contact-shell could equally have served as Phase 1 defaults, here we instead carried them into Phase 2, since each was tied to a specific mechanistic hypothesis about the system rather than used as a generic reference.

Phase 2.- Knowledge-driven customization

Using the structural context gathered in Phase 0 together with the results from Phase 1, expand the subselection space in targeted directions. Each new candidate subselection should correspond to a clear hypothesis about where the functionally relevant dynamics are expected to be encoded. A subselection restricted to a known allosteric interface, for instance, tests the hypothesis that inter-domain communication drives the functional distinction, while a subselection centered on a specific loop or motif tests a more localized hypothesis. This is the stage where system-specific expertise plays its largest role, translating the researcher's structural knowledge of the particular receptor-ligand system studied into concrete, testable atomic selections.

In our case this meant testing three further subselections against the MD2-backbone baseline: the backbone of the full receptor complex (TLR4+MD2), the all-atom polar rim (a region independently known to undergo conformational rearrangement upon binding), and the all-atom ligand-contact shell (residues within 5 Å of the ligand). Each encoded a distinct hypothesis about where the agonist/antagonist signal is carried: inter-domain allostery, a specific functional motif, and local recognition.

Phase 3.- Empirical refinement loop

This phase is what distinguishes the present approach from a purely knowledge-driven or purely data-driven strategy, and constitutes its main methodological contribution. Each candidate subselection generated in Phase 2 is evaluated not by assumption but by its effect on the GCNN's classification. A subselection is retained only if it yields a measurable and interpretable improvement over the Phase 1 baseline, or if it captures information that is demonstrably distinct from what the baseline already encodes. Subselections that do not meet this criterion should be discarded, or, if partially informative, folded back into a revised hypothesis for a further iteration of Phase 2.

This turns the choice of atomic subselection into a dynamic process rather than a one-time decision, and it is not limited to the choice of atoms alone: the same logic should be applied to other methodological choices that affect what the model actually learns, such as the simulation time window considered or the strategy used to split data into training and test sets. Any assumption that materially affects model performance should, wherever feasible, be treated as a hypothesis subject to this same empirical test rather than taken as a default.

In our case, classification accuracy varied substantially across the four candidate subselections: 97% for the TLR4/MD2 backbone, 92% for the MD2 backbone alone, 83% for the polar rim, and 79% for the ligand-contact shell, pointing to collective receptor dynamics rather than local ligand contacts as the main carrier of the agonist/antagonist signal. The same logic applied to the validation design itself: training on the full 150 ns trajectories gave no discriminative power (50% accuracy), while restricting to the 76-150 ns window raised accuracy to 92%, revealing that early equilibration dynamics were masking the functional signal.

Phase 4.- Robustness and validation check

Before a subselection is accepted as biologically meaningful, its apparent relevance must be distinguished from a modeling artifact. Two complementary checks are recommended:

- **Temporal validation:** Assess performance under a validation scheme that accounts for the temporal structure of the data, for example, comparing a random frame-based split against a split performed at the level of whole independent trajectories or replicas. Strong temporal autocorrelation within a single simulation can otherwise lead to inflated and misleading accuracy estimates.
- **Biological cross-check:** Wherever independent experimental or structural evidence exists, cross-check the regions or residues the model highlights as most relevant against that evidence. Convergence between an independent, performance-driven signal and orthogonal biological evidence provides considerably stronger support than either source considered alone.

In our study, splitting by whole trajectory rather than by random frame reduced accuracy from 92% to 51%, exposing strong temporal autocorrelation and showing that random-split accuracy alone could not be trusted as evidence of a generalizable signal. As a biological sanity check, several MD2 residues (e.g., Asn83, Lys122, Gly123) were consistently flagged as high-connectivity nodes across all four subselections and have independent mutagenesis support for a role in ligand binding, reinforcing confidence in the model-derived signal.

Final tips for adopting this methodology

The following recommendations synthesize the practical lessons of Phases 0 through 4 into a short set of guidelines for researchers applying this workflow to a new protein-ligand system:

- **Start coarse before going granular.** A highly specific, localized subselection is uninterpretable without a coarser baseline to compare it against. Always establish Phase 1 before attempting Phase 2.
- **Do not over-customize ahead of evidence.** Resist the temptation to build elaborate, knowledge-driven subselections before the empirical loop, Phase 3, has had the chance to test simpler hypotheses first.
- **Treat every methodological choice as a hypothesis, not only the atoms.** Validation strategy, time-window selection, and data-splitting procedure can all materially affect what the model learns and should be tested with the same rigor as the atomic subselection itself.
- **Guard against overfitting to a small ligand or replica set.** Report performance under both a random and a trajectory/replica-level split, since substantial divergence between the two is itself an important diagnostic signal.
- **Prioritize biological interpretability over raw performance.** A subselection that is difficult to map back to a physical or mechanistic explanation is of limited value, even if it achieves high classification accuracy.
- **Use convergence as the standard for relevance.** A region flagged as important across multiple, independently defined subselections, and, ideally, corroborated by external experimental evidence, should be trusted more than a signal obtained from a single subselection alone.
- **Document negative results.** Subselections that were tested and discarded are as informative to future users as those that were retained, and should be reported explicitly rather than omitted.
